# Channel-Type Induction Heating Tundish Technology for Continuous Casting: A Review

**DOI:** 10.3390/ma16020493

**Published:** 2023-01-04

**Authors:** Ziming Wang, Yue Li, Xiuzhen Wang, Xinlin Li, Qiang Yue, Hong Xiao

**Affiliations:** 1 School of Metallurgical Engineering, Anhui University of Technology, Ma’anshan 243002, China; 2 School of Mechanical Engineering, Anhui University of Technology, Ma’anshan 243002, China; 3 Hunan Zhongke Elect Co., Ltd., Electromagnet Ctr, Yueyang 414000, China

**Keywords:** tundish, induction heating, flow field, temperature field, magnetic field, inclusions

## Abstract

With the increasing demand for special steel, the quality of steel has become critical during the continuous casting tundish process. In recent years, tundish heating technology has played a key role in low superheat casting. Toward this, researchers have reported on the metallurgical effects of induction heating tundish (IHT). From 1984 to date, the channel-type IHT has been investigated in the production of continuous casting of special steel. In this article, the principle of this channel-type IHT technology and equipment composition were illustrated. A brief summary and comments were undertaken on the channel-type IHT, including physical modeling and numerical modeling. The application development trend of tundish induction heating equipment is summarized combined with industrial application data, which provide a reference for a better understanding of the induction heating process of tundish.

## 1. Introduction

Continuous casting tundish is an important vessel in the steel production process. Its technical development is significant to improve steel quality and production efficiency [1,2,3]. In the continuous casting process, the tundish is the last metallurgical vessel with refractory before the solidification of molten metal in the continuous casting mold. In addition to the two basic functions of molten steel storage and distribution, the tundish also plays a role in controlling the temperature and flow, promoting the inclusion removal [4,5]. The heat of molten steel is lost due to the various forms of heat exchange, leading to the temperature of molten steel dropping in the tundish, which affects the stability of the flow and temperature fields in the tundish [3,6,7,8]. In serious cases, the nozzle may be blocked. If the temperature of molten steel is too high, it will increase the temperature of molten steel in the process of converter steel tapping, increase the consumption of refractory materials for the steel ladle and tundish, reduce the casting speed of the continuous casting machine, and even cause mold breakout in serious cases. A too-low or high superheat of molten steel in the tundish will have a negative impact on the billet quality and the operation of the continuous casting machine [9,10,11,12]. Therefore, from the perspective of optimizing the whole continuous casting process, how to keep the temperature of molten steel within a reasonable range is important to realize the constant temperature pouring with low overheating in the tundish. It has become an urgent problem. Tundish heating technology has received more attention [13,14,15].

Tundish heating technology is important in tundish metallurgy, including plasma heating, high-temperature nitrogen heating, arc heating, and electromagnetic induction heating [7]. Among them, plasma heating and electromagnetic induction heating are the most widely used technologies. The comparison between tundish plasma heating technology and channel electromagnetic induction heating technology [4,16,17] is shown in Table 1.

Channel-type electromagnetic induction heating tundish (IHT) is developed by using the thermal and force effects of an electromagnetic field. This technology provides a new idea for realizing constant temperature-pouring for molten steel with low superheat and removing non-metallic inclusions from molten steel. The electromagnetic thermal effect can effectively compensate for the temperature of molten steel, reduce the temperature fluctuation of molten steel in the tundish, and achieve the goal of constant temperature pouring with low superheat in the tundish [18,19]. The electromagnetic force effect is reflected in the “pinch effect” generated by the electromagnetic field, which makes non-metallic inclusion particles with small density and poor conductivity move toward the channel wall, where they are finally removed [20,21]. Compared with tundish, and without an induction heating device, the IHT cannot only ensure the outlet temperature but also make use of the temperature difference to generate the density difference to make the molten steel have an upward trend in the distribution room, which is conducive to reducing short circuits and increasing the average residence time. Therefore, under the synergistic effect of the electromagnetic thermal effect and force effect, the molten steel in the channel-type IHT has a good temperature field and flow field, and inclusions are also easier to float and remove.

The tundish electromagnetic induction heating technology has been continuously developing with the progress of continuous casting technology and the improvement of steel quality requirements [4,22]. However, the theoretical and technical details of the technology are rarely reported due to technical confidentiality. The motion law of molten steel in the passage of electromagnetic IHT is still unclear under the actions of the external electromagnetic field. Due to the high temperature of molten steel and the short time for non-metallic inclusion aggregation or fusion in molten steel, hot state tests are not easy to carry out. In particular, under the thermal–mechanical coupling of the electromagnetic field, there is less research on the dynamics of inclusion aggregation and growth.

The non-isothermal flow of molten steel and the migration mechanisms of non-metallic inclusions are of great significance to purification and smelting under the electromagnetic field. In this paper, the basic principle, equipment composition, characteristics of molten steel, and non-metallic inclusions migration in the tundish under induction heating are systematically summarized. The development trend of the design and application of the tundish electromagnetic induction heating equipment are also discussed in order to provide a reference for a better understanding of the induction heating process of the tundish.

## 2. Principle of Electromagnetic Induction Heating

**Figure 1:**
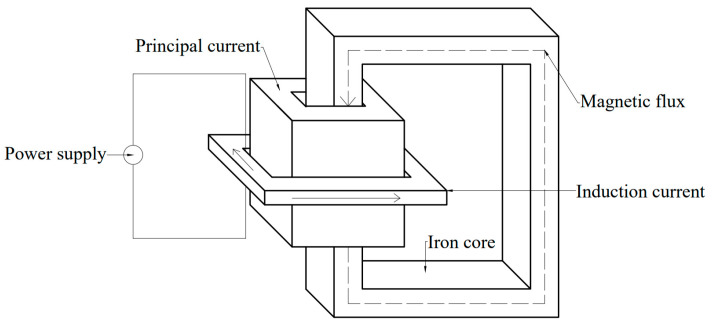
Schematics of the channel-type induction heating principle of the tundish [23].

Induction heating technology is based on the principle of electromagnetic induction. The field generator is installed in the tundish to make the molten steel produce the induced current when passing through the tundish channel. The molten steel is heated by Joule heat generated by the induced current. The schematic diagram is shown in Figure 1. The center of the heater induction coil is a closed-loop iron core, in which the iron core is surrounded by a primary loop. The molten steel channel and the steel flow in the tundish form a secondary loop. When the power frequency of the AC power is input to the coil, the alternating magnetic flux in the coil is generated. According to Faraday’s law of electromagnetic induction, the closed circuit composed of the alternating magnetic flux cutting molten steel will produce electromotive force *E* and Joule heat *Q* in the molten steel.

The expression of the electromotive force is as follows:

(1) $$E=\frac{d\phi }{dt}$$

The induced electromotive force *E* in the molten steel causes the induced alternating current *J* in the molten steel. The expression of the induced alternating current is as follows:

(2) J=σE

where *σ* is the conductivity of molten steel.

The Joule heat *Q* generated when the induced current passes through the molten steel can directly heat the molten steel in the channel and tundish. The expression of the Joule heating is as follows:

(3) $$Q=\frac{J^{2}}{\sigma }$$

The channel-type IHT is divided into the pouring chamber and distributing chamber (two parts) by a refractory baffle, and there is a channel connecting the two zones inside the baffle. The Joule heating of induction heating was generated under the actions of the induction current and molten steel resistance, mainly distributed in the channel. Molten steel flows through the channel and is rapidly heated by Joule heating. The molten steel heated by Joule heating is mixed with the molten steel in the distribution zone; the molten steel in the distribution area is also heated [24]. Compared with other tundish heating methods, the inductive current forms a circuit inside the molten steel, and the Joule heating generated by it directly compensates for the temperature of the molten steel in the molten steel; the heat is almost not dissipated. Therefore, the heating efficiency is higher than 90%, and the heating speed is faster [25].

The location of the electromagnetic induction heating device and how to cool the heating device are obstacles to the development of this technology. This restriction on the interior space size of the tundish limits the popularization and application of the electromagnetic induction heating technology of the tundish [25]. For this reason, many metallurgical workers are committed to improving the structures of tundish heating devices and channels.

## 3. Electromagnetic Induction Heating Device

**Figure 2:**
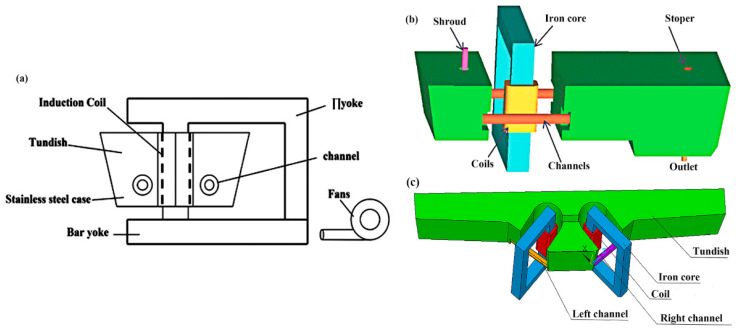
(**a**) Schematic of the tundish induction heating device [23]. (**b**) Tundish with a single induction heating device [26]. (**c**) Tundish with a single induction heating device [27].

The tundish electromagnetic induction heating device is mainly composed of an iron core, induction coil, cooling system, and power supply. Among them, the iron core and coil are the core of the electromagnetic induction heating device, constituting the induction heater. In order to facilitate disassembly and maintenance, the iron core is composed of upper and lower parts. The upper part is a Π-type iron core wound with an electromagnetic coil, which can be disassembled freely. The lower part is a bar yoke iron, which is fixed on the tundish. The upper and lower parts run through and are connected in the stainless-steel protective sleeve, which is mainly used for support, ventilation, and cooling. The cooling system mainly includes fan cooling and mist cooling. The heat generated by the device is taken away by the cooling medium to protect the equipment, which is safe and has high cooling efficiency. The channel is made of Al_2_O_3_-C refractory and set at the bottom of the tundish partition wall. Its main function is to heat the molten steel and introduce the molten steel flowing from the pouring zone to the casting zone. Its structure is shown in Figure 2.

The channel-type induction heater includes a single-coil induction heater and a double-coil induction heater. At present, the common induction heater mainly uses single-coil heating. During installation, the side with the coil is placed in the tundish and the side without the coil is placed outside the tundish. The single-coil induction heater is generally air-cooled. The cooling system is mainly composed of fans and pipes, and the non-magnetic stainless steel protective sleeve is also used as the cooling air channel. However, because the induction heating device itself occupies part of the space of the tundish, the liquid level drop speed of the IHT under the same casting speed is higher than that of the original tundish, resulting in more converging vortices at the tundish nozzle, slag entrapment, and suction, which seriously affect the quality of the slab. In order to solve this problem, a double-coil induction heater is proposed. This heater will not reduce the tundish capacity compared with the single-coil induction heater. In addition, the two-wire coil induction heater is water-cooled, and the deionized water is directly passed into the coil formed by winding the copper tube, which has high efficiency, small equipment, and no noise. However, most water-cooled equipment is made of copper, which has a certain impact on the electromagnetic induction heating effect [28]. Later, after continuous research and development, it became the current air mist composite cooling. The induction heater was externally cooled by air to cool the heat radiation around the induction heater. For the large eddy heat generated on the conductive tundish shell and coil, air mist cooling is used, which not only reduces the size of the heater cooling channel but also greatly reduces the volume of the cooling system, greatly ensuring the safety of users [29].

The influence of the power, size, and other factors of the device on the stability of the tundish should be comprehensively considered when selecting devices due to the different types of tundishes and the characteristics of the channel design. At present, channel-type IHT is widely used, but the added induction coils and channels occupy the effective volume of the tundish, which can be optimized in the future tundish reconstruction design.

## 4. Physical Field and Inclusion Characteristics in IHT

### 4.1. Magnetic Field of Tundish with Channel Induction Heating

Electric magnetic fields play a critical role in regulating molten steel flow, heat transfer, and inclusion migration [26]. Compared to receiving and distributing chambers, the channels have the highest-induced current densities, magnetic flux densities, electromagnetic forces, and Joule heating, and they are symmetrically distributed [27,30,31]. One-third of the total inclusion removal rate in the tundish is associated with the channel. If channel induction heating is applied, the inclusion removal increases from 21.4% to 35.05% in the tundish [32]. Therefore, the channel is an important place for inclusion removal.

**Figure 3:**
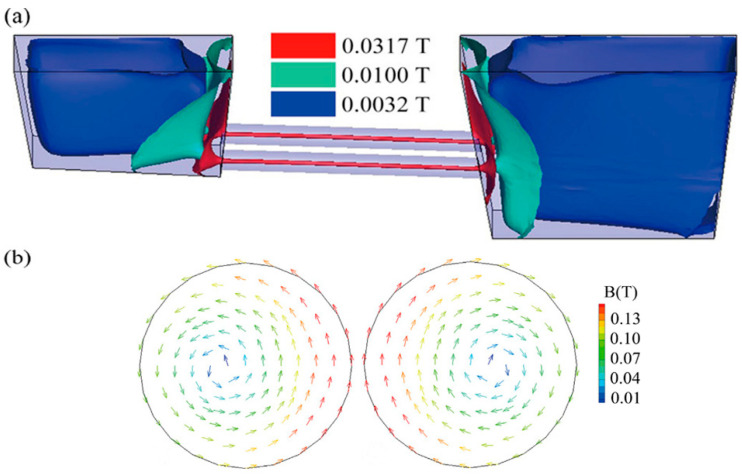
Distribution of magnetic field in the tundish [33]. (**a**) The effective area of the electromagnetic field in the chamber, (**b**) the vector of the magnetic flux density in the channels.

Figure 3a shows the simulation results of the effective area of the electromagnetic field in the chamber. It can be seen from the results that the effective active zone of the magnetic field contains the channels and the nearby walls connected with the channels [33,34]. Skin effect and proximity effect lead to asymmetric distribution of the electromagnetic force near the channel outlet [27]. It is obvious that channel outlets have stronger electromagnetic forces in the upper area. The result is the downward flow of the mainstream, and the trajectory of inclusions is altered. Figure 3b shows that the magnetic flux density is rotated on the cross-section of the channel.

The magnetic induction intensity and current density generated by molten steel in the channel increase linearly with the increase of the coil’s current intensity. With the increase in the current frequency, the induced current density on the channel surface and Joule heating generated by molten steel increase continuously, while the magnetic induction intensity and electromagnetic force decrease.

**Figure 4:**
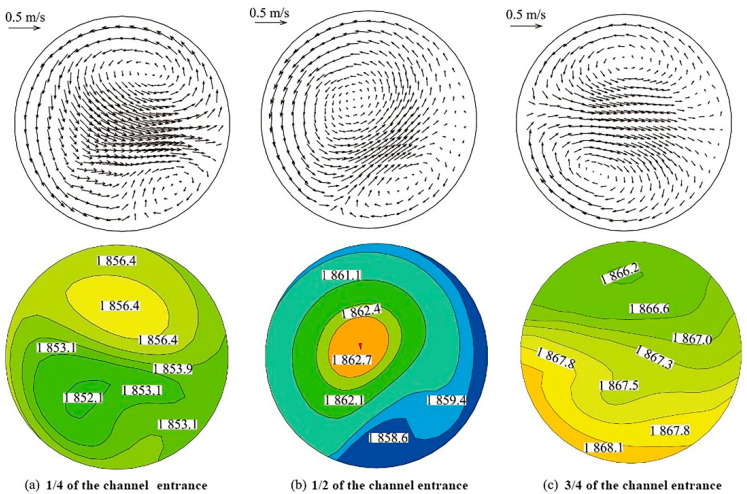
Molten steel flow and heat transfer at the different positions of the IHT channel [37].

The induced current is mainly distributed in the channel close to the induction coil and two chambers, and the current forms a current loop through the channel. The magnetic flux density of the channel close to the induction coil and the two chambers is far greater than that of other areas, so the magnetic field in the channel is an eccentric rotating magnetic field, and the electromagnetic force is an eccentric force pointing to the center of the channel [27,35,36]. On the one hand, inclusions are significantly removed under this electromagnetic pressure, especially for larger inclusions. Due to the existence of the electromagnetic field, the molten steel in the channel has a rotating speed under the action of the eccentric electromagnetic force, which changes from the direct current to the spiral flow, and its flow mode is composed of two counter-rotating eddy currents near the outlet area of the channel [7,24,37]. The molten steel in the channel presents an alternating flow between the single vortex and double vortex with time, which will likely aggravate the channel erosion [38,39,40,41]. Joule heat distribution generated under the action of the induced current and molten steel is basically the same as that of the induced current. After induction heating in the channel, the molten steel tends to flow upward under the action of buoyancy, which reduces the short circuit flow and extends the residence time [39]. Figure 4 shows that molten steel flow and heat transfer at the different positions of the IHT channel. The diameter of the channel pipe is 140 mm, showing double eddy currents in the cross-section. Due to the uneven Joule heat distribution, the temperatures (Kelvin temperature) of different cross sections are different.

The research studies on the electromagnetic field in the channel-type IHT not only provide important theoretical bases for the development of this technology but also greatly enrich the research on the application of electromagnetism in the tundish metallurgy. However, in view of the different physical media used to simulate the flow and heat transfer of molten steel, we also need to more comprehensively consider the influences of the physical and chemical properties of the simulation media on the simulation results. This is of great significance for verifying the thermodynamic coupling model in the electromagnetic field. The flow characteristics of molten steel in the channel change with time and further attention should be paid to the transient flow characteristics of molten steel in a non-isothermal state under the actions of the electromagnetic field, especially in the traditional cold state simulation. The physical field and inclusion characteristics of molten steel in the tundish are different with or without induction heating.

### 4.2. Flow and Heat Transfer in Tundish with Channel Induction Heating

The flow field of molten steel in the tundish will not only affect the removal of inclusions but also greatly affect the temperature uniformity of molten steel. In order to improve the tundish’s performance, it is important to focus on the flow behavior of the tundish [42]. At present, most tundishes are optimized by installing turbulence controllers or devices, such as dams and weirs. The combination of upper and lower weirs can also effectively improve the flow field in the IHT; moreover, the dead zone volume in the tundish will be reduced, the average residence time of molten steel in the tundish will increase, and the short circuit flow phenomenon is basically eliminated [43].

In the multi-flow tundish flow control device, the V-shaped retaining wall with the pilot hole, weir, dam, and turbulence inhibitor are well employed together [44]. Compared with the V-shaped retaining wall, the combination of the inverted splayed weir wall and circular turbulence suppressor is better. It can better improve the flow field and temperature field of the four-flow H-type dual-channel IHT. The speed distribution in the entire pouring area is uniform, the average speed is small, and the free surface speed is small, which is conducive to improving the residence time of molten steel, promoting the floating of inclusions, reducing the erosion of refractory materials, and reducing the tendency of slag entrapment [45].

In addition to installing flow control devices, many people began to improve the structure of the induction heater. The common double-induction heater takes up more tundish capacity, making the effective volume smaller, resulting in the reduction of the steel plant’s output. Therefore, a new type of trough–single induction heating pouring tundish was proposed, and its flow characteristics were calculated by a water model experiment. The results show that the dead body’s integral and the shortest interruption time of the tundish are much better than those of the double induction heating furnace, but the flow consistency between different strands of the single induction heating furnace is still slightly weaker than that of the double induction heating furnace [46]. The consistency of each flow is a concern of multi-flow tundish metallurgy optimization. The large temperature differences at each outlet and the superheat of molten steel casting are common problems in optimizing IHT.

Based on this, a split channel scheme [47,48,49,50] is proposed, which reduces the proportion of the dead zone, extends the average residence time of molten steel, and reduces the temperature difference of each flow in the tundish. The flow uniformity of the whole tundish can be significantly improved with the dam structure, the residence time distribution (RTD) curves of each nozzle almost coincide, and the industrial test results are better than the traditional straight channel. The overall average residence time of the tundish is prolonged, the proportion of the dead zone is reduced, and the consistency of each flow greatly improves after adding double dams on the basis of the use of split channels compared with the prototype tundish [51]. Due to the asymmetry of the multi-stream tundish, the distance between each outlet and the tundish inlet is different. This makes the average residence time of each outlet different, and there is a large temperature difference at each outlet. When the channel is provided with a side hole, the flow stream flowing to the middle of the tundish through the side hole gradually increases, which is conducive to enhancing the fluidity of the middle region.

As the refractory materials are continuously eroded by the molten steel, the refractory particles will be brought into the molten steel, resulting in secondary pollution of the molten steel [52]. Whether it is a turbulence controller or a dam, it will occupy the inner space of the tundish, and there will be a dead zone behind the weir, which will shorten the residence time of the molten steel.

The thermal convection is caused by Joule heating, and the flow pattern of molten steel significantly improves under the horizontal magnetic field or vertical magnetic field. The temperature fluctuation of molten steel at the outlet is reduced. At the same time, the removal rate of non-metallic inclusions in molten steel is improved [53]. The stirring effect of the electromagnetic force on molten steel is the main factor affecting the flow pattern, while the thermal convection caused by Joule heating is the second factor affecting the flow pattern [32,54,55,56].

**Figure 5:**
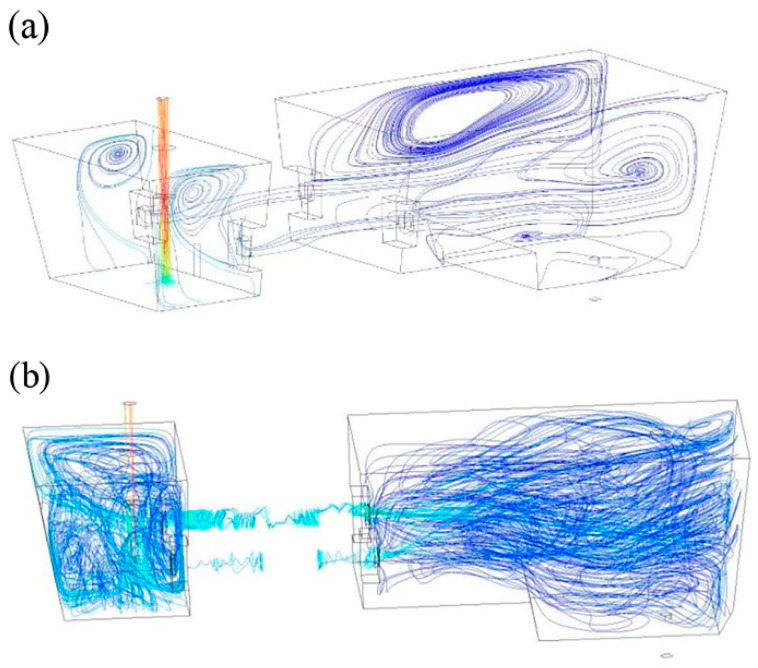
Molten steel path line in tundish without (**a**) and with (**b**) induction heating [26].

In Figure 5, a path line for molten steel with and without induction heating is shown. As seen in Figure 5, under the induction heating condition, the molten steel is subjected to the centripetal electromagnetic force and Joule heating flows in a spiral shape in the channel. Therefore, the molten steel still has a large rotation speed when flowing out of the channel, and it flows to the tundish outlet. Compared with no induction heating, under the action of the electromagnetic field, the rotation of molten steel in the channel is strengthened, and the tangential velocity of molten steel in the channel increases, which is conducive to increasing the average residence time of molten steel and removing non-metallic inclusions in molten steel [26].

Thus, it can be inferred that using water at different temperatures to simulate the electromagnetic induction heating process, ignoring the electromagnetic force of molten steel, and using the water model to simulate the non-isothermal movement of molten steel is incomplete [24]. Conventional physical simulation can still be used as an important basis for structural optimization in the promotion and application of new IHT technology. However, the strength of natural convection due to temperature differences, the pinch effect of the electromagnetic force on molten steel, and the new requirements for channel refractory still depend on the analysis of the numerical model.

A numerical simulation is an effective tool to analyze complex metallurgical multiphase flow problems. In the absence of high-temperature melt experiments, numerical simulation is still one of the important methods used to study the state and inclusion characteristics of molten steel in tundish under non-isothermal conditions [42,57]. Many metallurgical workers have studied and verified the relationships between the characteristics of physical fields and the inclusions of tundish and the heating power, channel structures, tundish flow control devices, and other factors under induction heating based on numerical simulations. Some people verified the optimization effect of induction heating on the temperature field and flow field of the tundish through numerical simulations. The calculation results showed that the temperature of molten steel in the tundish significantly increased, the overall average residence time extended, and the overall dead zone volume fraction was reduced by using induction heating [55,58].

The heating power plays an important role in the temperature and uniformity of molten steel in the tundish. The flow of molten steel in the channel and the pouring chamber was active, and the swirling flow was generated in the channel. With the increase of the heating power, the temperature-rising speed of molten steel in the tundish became faster and the temperature field distribution was relatively uniform. The tangential velocity of the channel section significantly increased, and the rising flow was generated in the pouring chamber, which extended the average residence time and reduced the dead zone volume fraction and the occurrence of the short circuit flow [35,54,59,60].

**Figure 6:**
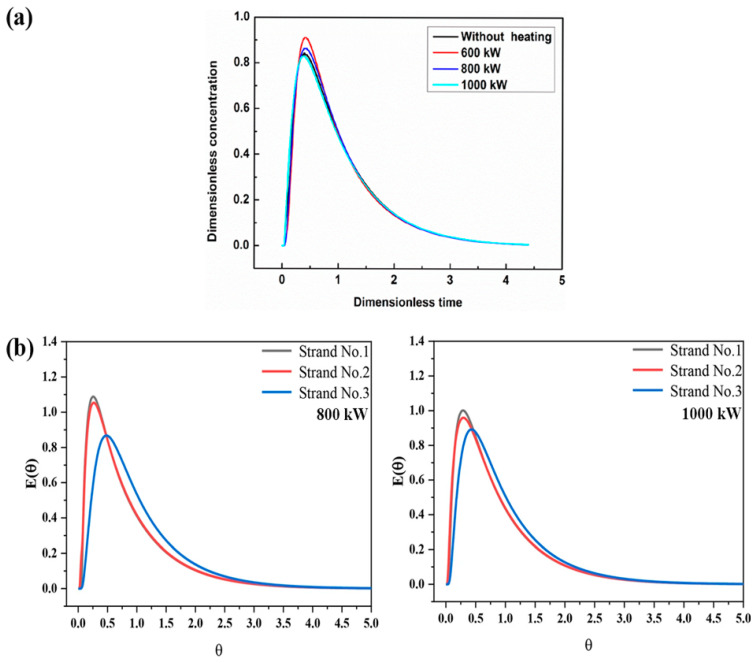
RTD curves in the tundish with different types of heating of power. (**a**) The single-flow dual-channel IHT [61]. (**b**) The dual-channel IHT [54] © The Minerals, Metals, & Materials Society and ASM International 2021. All rights reserved.

However, the higher heating power is not always better. For the single-flow dual-channel IHT whose capacity is 25 tons, when the heating power is in the range of 600~800 kW, with the increase of the heating power, the temperature distribution in the casting area becomes uniform, the temperature stratification in most areas is basically eliminated, and the total inclusion removal efficiency is improved. Compared with 600 kW, the tundish performs better on parameters such as resident time, dead volume, and the ratio of the plug to dead volume when using 800 kW heating power, as shown in Figure 6a. However, when the heating power exceeds 800 kW, the flow field of the tundish becomes chaotic and the uniformity of the temperature field is worse. Therefore, the reasonable heating power of the single flow channel IHT should be controlled at 600~800 kW [60,61]. Others found that when the heating power is 1000 kW for the six-strand dual-channel IHT, not only is it beneficial in compensating for the heat loss from the tundish, reducing the dead zone rate, but also for improving the consistency of the flow and the temperature, greatly improving the internal quality of the billet and the stability of the final product [54], as shown in Figure 6b.

The optimal heating power is not the same under the different tundish types; therefore, the channel design and arrangement of the tundish also have a great influence on the flow and heat transfer in IHT. Compared with the common straight channel, the curved channel can make temperature distribution more uniform in the casting zone by changing the flow direction of molten steel at the outlet. The IHT with the curved channel has faster heating efficiency, higher efficiency, and can better avoid the formation of a dead zone [62]. The influences of different channel radii on the flow, heat transfer, and inclusions in IHT have also been studied. Some researchers [63] believe that a larger channel is conducive to the inclusion growing in the cavity. The larger the channel diameter, the larger the macroscopic mixing effect, the higher the inclusion removal rate, and the smaller the characteristic inclusion radius at the tundish outlet. Therefore, a channel with variable diameters was proposed [47,64]. The flow field is optimized and the temperature uniformity is improved after adopting this channel.

**Figure 7:**
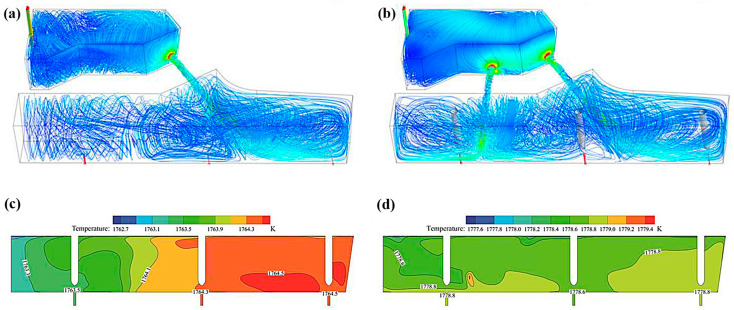
The 3D streamline patterns of molten steel in the tundish and the temperature distribution in the longitudinal section through the outlets of each strand; (**a**,**c**) are the double channel; (**b**,**d**) are four channels [66]. © 2022 Wiley-VCH GmbH. All rights reserved.

Some people believe that the use of a dual channel can reduce the maximum temperature difference between each share and effectively reduce the dead zone ratio in the tundish, and the effect will be more significant after increasing the dam body [65] compared with the single channel. Under the same ampere turns, the joule heat generated in each channel is basically the same, whether in the dual-channel tundish or the four-channel tundish. Therefore, the more channels, the higher the total heat generated and the higher the heating efficiency. Under the same induction heating conditions, the temperature of the four-channel structure can be 1.7 times higher than that of the dual-channel structure, and the maximum temperature difference in the tundish is much smaller. It can be seen from the simulation results in Figure 7 that, compared with the dual-channel tundish, the flow field of the four-channel tundish is better, which can significantly improve the fluid flow control and reduce the scouring of the channel sidewall; the standard deviation of the average residence time is also lower. Moreover, the temperature of the four-channel tundish is more uniform, and the consistency of the outlet temperature is better than that of the dual-channel tundish [66].

The obliquity and height of the channel also have certain effects on the flow and heat transfer in the tundish. The change of the channel’s inclination angle can alleviate the upward impact force of molten steel to a certain extent, but expand the range of the low temperature zone. The turbulence intensity of molten steel in the pouring area decreases as the inclination angle increases, which is beneficial to the uniform composition and temperature of molten steel [67]. However, when the channel outlet is lower than a certain value and the dead zone volume of the tundish is kept within a certain range, changing the channel angle and setting the dam body have little effect on the fluid flow characteristics, while raising the channel outlet and adding double dam structures on both sides of the tundish can effectively optimize the flow field and temperature of the IHT [68,69,70].

### 4.3. Inclusions in Channel IHT

The electromagnetic force can significantly promote the removal of small inclusions, it also has a good effect on reducing the total oxygen content in molten steel [33]. The principle of separation (of the inclusions) is that the electromagnetic force presses the metal liquid toward the axis of the pipe, thus producing a pinch effect. This force generates a pressure gradient in the melt, making the force field around the inclusions unbalanced so that the non-metallic inclusion particles in the metal liquid move toward the pipe wall under the electromagnetic pinch force. Finally, it is attached to the pipe wall and removed [71,72]. The temperature of molten steel flowing through the channel increases and the density decreases under the action of induction heating, which is another factor that promotes inclusion removal. The metal liquid forms an upward flow field after flowing out of the channel, which promotes Stokes’ collision, the accumulation, and growth of inclusions, and improves the adsorption and removal efficiencies of the tundish slag [73].

**Figure 8:**
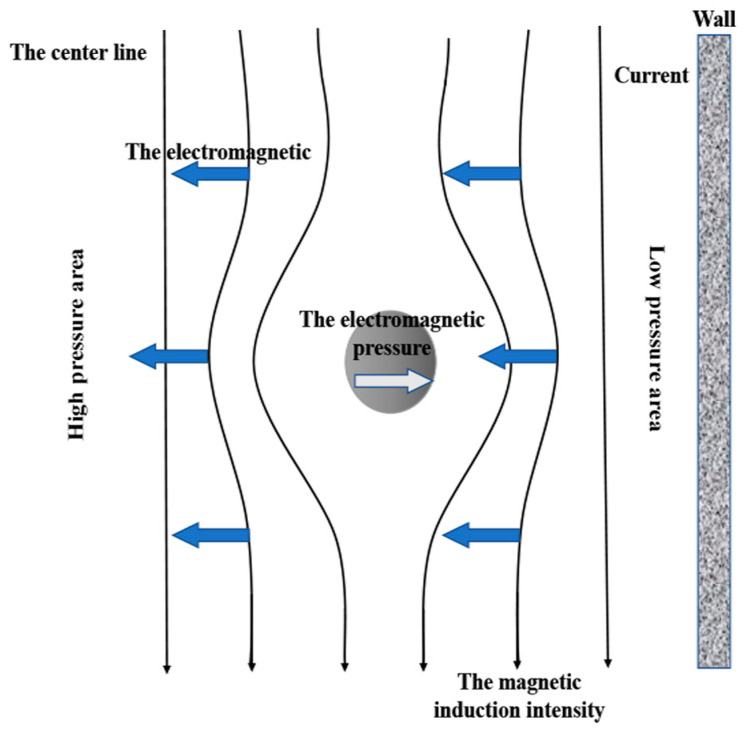
Schematic of electromagnetic pressure [74].

When induction heating is applied, the molten steel temperature increases sharply, and thermal buoyancy can increase the average residence time of molten steel in the tundish, which is conducive to the floating of the inclusion. As mentioned earlier, under the pinch effect, the pressure difference distribution of molten steel promotes the movement of inclusion toward the wall, adsorption, and removal, especially for the small inclusions [24,74], as shown in Figure 8. This trend increases with the increase in heating power. On the other hand, the electromagnetic force also can stir the molten steel, completely mixing it in a short time, increasing the mixed zone fraction. Melt mixing is also conducive to collision coalescence and the removal of inclusions [33,56]. When the molten steel enters the distribution chamber, no matter the particle size, about 75% of the inclusions will be adsorbed by the tundish covering flux, and the adsorbed inclusions will almost float to the free surface within 120 s [33].

It can be seen that the force effect of electromagnetic induction is key to controlling the change of molten steel flow [54,75]. When the inclusion is in the lower half of the circular channel, under a certain magnetic field strength, the electromagnetic force on the inclusion and its own buoyancy will reach equilibrium, and there will be a dead zone near the equilibrium position. At this time, the removal time of the inclusion under the action of the electromagnetic force is longer than that without the magnetic field. The range of the dead zone decreases with the increase of the magnetic field strength. Therefore, the dead zone can be reduced by increasing the magnetic field strength, thus promoting the movement, collision, and removal of inclusions [76].

**Figure 9:**
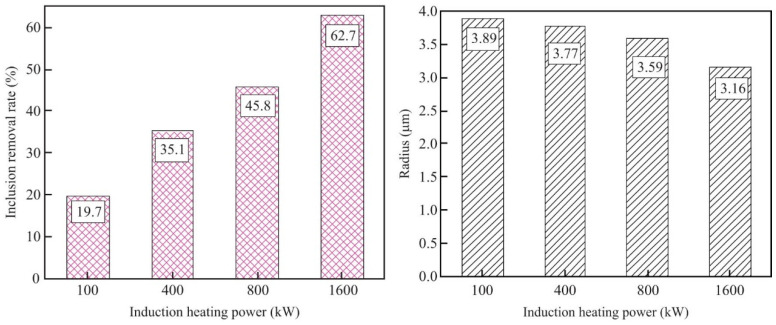
Inclusion removal rate of IHT with different types of heating power [63].

Power ampere turns have significant impacts on the magnitude of an electromagnetic force and magnetic field strength. During induction heating of the tundish, the electromagnetic force on the central axis of the channel increases with the increase of power ampere turns [27]. The removal rate and escape rate of inclusions fluctuate sharply when the heating power is altered by comparing the removal rate of inclusions under continuous heating and intermittent heating [59]. When the induction heating power continues to increase, the removal rate of inclusions also continues to increase [39]. Therefore, the removal rate of inclusions increases, and the characteristic radii of inclusions at the outlet decrease with the increase of the induction heating power [63,74]. Figure 9 shows the inclusion removal rate and the characteristic inclusion radius of the tundish with different induction heating. When the induction heater has higher power, a stronger electromagnetic force is generated, which allows for the inclusion of the tundish to be removed more quickly. The larger induction heating power generates a larger electromagnetic force and turbulent kinetic energy so that the small inclusions have more chances to collide, coalesce, and float to the slag layer.

However, when electromagnetic forces are excessive, violent turbulent flow can occur in the tundish. The molten steel will be heated rapidly under the joule heating; it rotates and flows under the action of an electromagnetic force. It would aggravate the erosion of refractory materials and affect the cleanliness of molten steel. Hence, it is necessary to select the appropriate induction heating power according to the production requirements of industrial applications [63].

### 4.4. Industrial Application of Channel-Type IHT

In 1984, a patent for channel-type IHT was proposed for heating the molten steel in the tundish [77,78]. The IHT compensates for the temperature drop of molten steel in the tundish by the electromagnetic induction heat effect. The experimental results show that the temperature of molten steel decreases to 0~5 °C when the tundish is heated by induction, while the temperature of molten steel decreases to 10~20 °C when the tundish is not heated by induction. The temperature control accuracy is ±3 °C, and the thermal efficiency is 70~100%, with an average of 85%. Subsequently, a single-channel tundish induction heating device was proposed [78]. In 1987, a double-channel tundish induction heating device was designed with temperature control up to ±2.5 °C. Subsequently, various shapes of IHT have been designed.

At present, IHT technology is adopted by steel plants [25], for example, the Kawasaki Company of Japan uses an arc single-channel IHT to produce SUS304 steel and 430 steel, which can control the temperature accuracy at ±2.5 °C. Nippon Steel not only carried out temperature control after using the parallel dual-channel IHT but also successfully completed the experiment of an online adjustment of molten steel composition; they achieved good results [79]. The application examples are shown in Table 2.

The influence of induction heating on tundish and continuous casting tundish was simulated under the conditions of molten steel flow and thermal effects with induction heating by Zhao [80]. The results show that the temperature gradient of electromagnetic induction heating is small and the heating effect is more uniform. In different conditions, such as heating power and casting speed, the temperature field in the tundish has no obvious change, and the heating efficiency is as high as 80.7%. An IHT with a variable cross-section heating groove was developed; it speeds up the heat exchange, improves the thermal efficiency, and uses new refractory materials to prolong the service life of the tundish. Subsequently, a by-pass dual-channel electromagnetic refining and heating device and a cross-type tundish channel induction heating device were introduced; they have good heating efficiency and long average residence times of molten steel, thus promoting the upward removal of inclusions in molten steel. The transverse side groove electromagnetic IHT makes it applicable to various types of tundish structures. Continuous optimization of the advanced technology of the channel-type electromagnetic IHT by metallurgical workers makes this technology more widely used in practical production.

In recent years, the square billet of special steel mainly involves multi-strand continuous casting with increasingly high requirements for steel quality, especially the steel produced by special processes and requiring special properties. The control requirements for the consistency of the flow and temperature of each strand in the tundish are very high in order to ensure smooth production and consistent billet quality. Induction heating can effectively compensate for the temperature, reduce the superheat of molten steel pouring, and ensure the consistency of temperature of each strand in the continuous casting process. The tundish induction heating technology not only does not need to change the existing continuous casting process but also helps to adjust the steel composition online and improve the central segregation. Therefore, it is particularly effective for the production requirements of multiple varieties, small batches, and continuous casting of different steel grades in industrial production, which are very suitable for application and promotion in existing continuous casting productions.

**Figure 10:**
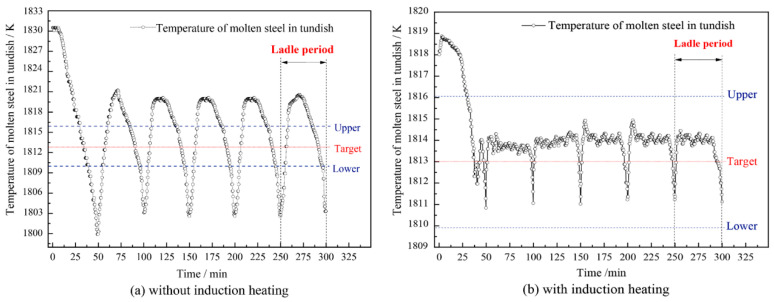
Molten steel temperature in tundish without (**a**) and with (**b**) IH [26].

The temperature loss of molten steel in the tundish is great at the end of pouring (as with the tundish without an induction heating device). At this time, priority is given to heating compensation; induction heating operates at a high power to achieve the constant temperature casting of continuous casting, improve the quality of continuous casting billets, and reduce the cost of the production process [81]. As shown in Figure 10, the temperature fluctuation of molten steel in tundish can be controlled within ±5 °C by adjusting the heating power, which is conducive to realizing stable casting with a low superheat under electromagnetic induction heating. The temperature of molten steel in the tundish without induction heating fluctuated within ± 20 °C, which is not conducive to the smooth progress of continuous casting [37].

IHT with a splayed channel has the advantages of non-contact heating, good responsiveness, high heating efficiency, good purification effect of molten steel, good automatic control, and easy installation and maintenance. The splayed channel IHT has been successively used in steel plants. The industrial test results showed that the metallurgical results were satisfactory. The temperature drop of molten steel in tundish during the whole casting process was studied by using continuous temperature measurements and induction heating. It was found that the temperature drop rates at the end of the second and third furnaces in the middle of the pouring furnaces were relatively high. The target superheat can be controlled within the range of 15~20 °C, adopting induction heating technology to compensate for the tundish temperature.

With the improvement of automation and reliability of the power supply, induction heating equipment will have wider application prospects. At present, industrial applications mostly adopt heating under fixed power supply power and frequency, which will reduce the temperature differences inside and outside the channel, which is not conducive to maintaining the appropriate temperature differences. In the future, the negative feedback on dynamically controlling the power supply can be included in the optimization directions of induction heating devices. The induction heating system is progressing toward intelligent control. In the future, the induction heating system with computer intelligent interface, remote control, automatic fault diagnosis, high efficiency, and energy-saving control performance will be expanded to more fields.

## 5. Conclusions and Prospect

Channel-type IHT technology is of great significance to improve the production efficiency and billet quality of the continuous casting. The results of numerical simulation, physical modeling, and industrial production show that induction heating technology in the tundish is an effective means of clean steel production.

(1) Channel-type IHT effectively compensates for the temperature drop of molten steel in the tundish to make the temperature field distribution in the tundish uniform, effectively optimizes the flow field in the tundish, promotes the removal efficiency of inclusions in the molten steel, and improves the cleanliness of the molten steel.

(2) Fluid motion and inclusion migration should be considered in order to investigate the flow and heat transfer of liquid metal under an electromagnetic field in a high-temperature experiment. The molten steel in the channel flows under the combined action of the force and thermal effects of an electromagnetic field. The mechanisms of inclusion aggregation and adhesion to the wall need to be further investigated in high-temperature experiments.

(3) The molten steel pinches when it flows through the channel, which makes the molten steel in the channel pulsate and leads to an unstable flow field in the tundish under the action of the electromagnetic field. The greater the heating power, the stronger the disturbance. In industrial production, different types of heating power should be selected in different pouring stages.

(4) The temperature fluctuations of molten steel in the tundish without induction heating are within ± 20 °C, while under an electromagnetic induction heating condition, the temperature fluctuation of molten steel in the tundish can be controlled within ± 5 °C.

## Figures and Tables

**Table 1:** Comparison between tundish plasma heating technology and electromagnetic induction heating technology.

Comparison Items	Plasma Heating	Induction Heating
Heating power and efficiency	Power: 1000 kw, efficiency about 65%	Power 1000 kw, efficiency ≥ 90%
Temp accuracy	±5 °C	±2~3 °C
Pollution	Increase nitrogen content in molten steel	No pollution
Environmental effect	High noise and electromagnetic radiation	No noise and low electromagnetic radiation
Operational requirements	Complex and demanding operation	Simple operation
Equipment maintenance	Frequent replacement of cathode materials is required	Less maintenance
Operating expenses	High cost	Low cost

**Table 2:** The application case of channel-type IHT technology [79].

Steelworks	Tundish Capacity/t	Steel	Heating Power
Kawasaki Heavy Industries, Ltd.	8	SUS304, 430	1000 kW
Daido Steel Co., Ltd.	20	Low Alloye Steels, Bearing Steel	1000 kW
SUMITOMO SHOJI KAISYA, LTD.	13	High Carbon Steel, Bearing Steel, Carburized steel	1000 kW
Nippon steel corporation	30	low silicon aluminum killed steel	1000 kW
Kobe Steel., Ltd.	12	Bearing Steel	600 kW

## Data Availability

The data that support the findings of this study are available upon request from the corresponding author.
